# Vitamin E Is a Nephroprotectant Agent in Male but Not in Female in a Model of Cisplatin-Induced Nephrotoxicity

**DOI:** 10.5402/2013/280395

**Published:** 2013-06-23

**Authors:** Sima Jilanchi, Mehdi Nematbakhsh, Mehrnoosh Bahadorani, Ardeshir Talebi, Fatemeh Eshraghi-Jazi, Azam Mansouri, Farzaneh Ashrafi

**Affiliations:** ^1^Water & Electrolytes Research Center, Isfahan University of Medical Sciences, Isfahan 81745, Iran; ^2^Deparment of Biology, Falavarjan Branch, Islamic Azad University, Isfahan 84515, Iran; ^3^Department of Physiology, Isfahan University of Medical Sciences, Isfahan 81745, Iran; ^4^Kidney Diseases Research Center, Isfahan University of Medical Sciences, Isfahan 81745, Iran; ^5^Department of Clinical Pathology, Isfahan University of Medical Sciences, Isfahan 81745, Iran; ^6^Department of Internal Medicine, Isfahan University of Medical Sciences, Isfahan 81745, Iran

## Abstract

*Background*. The role of gender for nephroprotectant agent such as vitamin E in cisplatin- (CP-) induced nephrotoxicity has not been documented yet. *Methods. *One group from each gender of Wistar rats received a single dose of CP (7 mg/kg; i.p) and was treated with vitamin E (1 g/kg/day) for 7 days, and they were compared with similar gender in the control group. *Results*. The serum levels of blood urea nitrogen (BUN) and creatinine (Cr) in male animals treated with CP was not different from the control group, but it was significantly different in the female rats (*P* < 0.05). The CP-induced damage intensity in male kidney tissue was not significantly different between the CP-treated and control groups, but this was not the case in female, indicating that the tissue damage in female is significantly different from the control group (*P* < 0.05). No significant difference in serum levels of magnesium (Mg), nitrite, malondialdehyde (MDA), and lactate dehydrogenase (LDH) was seen between the genders. Kidney weight and body weight changes were statistically significant in both genders (*P* < 0.05). Significant difference was observed in uterus weight between the two groups of female (*P* < 0.05). *Conclusion*. Vitamin E may prevent CP-induced nephrotoxicity in male, but possibly it has not such nephroprotectant effect in female.

## 1. Introduction

Cisplatin (CP) is an antitumor drug widely used in clinic [1]. This drug contains heavy metal platinum [2] and is used to treat a variety of neoplasms such as head, neck, testicular, ovarian, bladder, small cell lung, and esophagus cancers [3]. CP therapy produces free radicals such as superoxide and hydroxyl [4, 5] that lead to oxidative stress followed by nephrotoxicity, neurotoxicity, hepatotoxicity [2, 6], and endothelial dysfunction [7, 8]. Kidney dysfunction causes glutathione depletion [9], decreases creatinine (Cr) clearance [10], and decreases the glomerular filtration rate [11, 12]. To prevent CP-induced nephrotoxicity, renin angiotensin receptor antagonist (losartan) and different antioxidant substances have been suggested to be supplemented. L-arginine is the main precursor of nitric oxide (NO) in vascular endothelium, and its renoprotection role against CP-induced nephrotoxicity was reported [13]. The effects of vitamins C and E on renal injury induced by CP have been reported previously. Vitamin C reduces free radicals in many biological processes [14] and protects the kidney against CP-induced oxidative stress [14]. Vitamin E, as a membrane stabilizer, is well known as free radical scavenger [15], and its nephroprotective role against CP-induced nephrotoxicity was previously reported [16–18]. Recently, the sex-based difference in kidney toxicity was the subject of researches [17, 19, 20]. Losartan as an angiotensin receptor-1 blocker and L-arginine provides different roles against CP-induced nephrotoxicity in male and female rats [21, 22]. One study showed that vitamin E has no protective role against CP induced nephrotoxicity in ovarectomized female rat when pharmacological dose of estrogen was administrated [23]. Also, it has been reported that vitamin E has renoprotective effect against CP-induced nephrotoxicity in male rats, but the role of gender for nephroprotectant agent such as vitamin E in cisplatin (CP)-induced nephrotoxicity has not been reported yet [24, 25], Thus, we attempted to investigate the nephroprotective role of vitamin E in male and female rats.

## 2. Materials and Methods

### 2.1. Animals

Twelve adult female (weighting 177.7 ± 2.4 g) and fourteen adult male (weighting 199 ± 3 g) Wistar rats (Animal Center, Isfahan University of Medical Sciences, Isfahan, Iran) were used for this research. The rats were housed at the temperature of 23–25°C. Rats had free access to water and rat chow. The rats were acclimatized to this diet for at least one week prior to the experiment. The experimental procedures were in advance approved by the Isfahan University Medical Sciences Ethics Committee.

### 2.2. Drugs

Vitamin E and CP (cis-diammineplatinum(II) dichloride, code p4394) were purchased from Sigma (St. Louis, MO, USA).

### 2.3. Experimental Protocol

Wistar rats were randomly assigned to four groups. The male animals in group 1 (*n* = 7) received a single dose of CP (7 mg/kg) and then were treated with vitamin E (1 g/kg/day) for 7 days. The male animals in group 2 (*n* = 7) received the same regimen as group 1, except vehicle (saline) instead of CP. Female animals in groups 3 (*n* = 6) and 4 (*n* = 6) received regimen the same as groups 1 and 2, respectively. The weight of animals was recorded on a daily basis. One week after CP injection, all animals were sacrificed after blood sampling. The serum was stored at −20°C for the measurements. The kidneys and testis (or uterus in female) were excised and weighed immediately. The left kidney was used for histopathological investigations, and the right kidney was homogenized in 2 mL of saline and then centrifuged, and the supernatant was used for measurements.

### 2.4. Measurements

Serum levels of creatinine (Cr), blood urea nitrogen (BUN), lactate dehydrogenase (LDH), and magnesium (Mg) were determined using quantitative diagnostic kits (Pars Azmoon, Iran). The serum level of nitrite (stable NO metabolite) was measured using a colorimetric assay kit (Promega Corporation, USA) that involves the Griess reaction. Serum and kidney levels of malondialdehyde (MDA) were quantified according to the manual method. Briefly, 500 *μ*L of the sample was mixed with 1000 *μ*L of 10% trichloroacetic acid (TCA). The mixture was vigorously shaken and centrifuged at 2000 g for 10 min; 500 *μ*L of the supernatant was added to 500 *μ*L of 0.67% thiobarbituric acid (TBA). Then, the solution was incubated for 10 min in warm water bath at the temperature of 100°C. After cooling, the absorbance was measured at the wavelength of 532 nm.

#### 2.4.1. Histopathological Procedures

For histopathological investigation, the excised left kidney was fixed in 10% formalin solution, then was embedded in paraffin. The hematoxylin and eosin stain was used for assaying the tubular damage. Parameters of tubular damage included tubular dilation and simplification, tubular cells swelling and necrosis, tubular casts, and intraluminal cell debris with inflammatory cells infiltration that were considered in the pathological study. The intensity of tubular injuries was graded from 1 to 4, while zero score was assigned to normal tissue without damage.

#### 2.4.2. Statistical Analysis

Data are presented as mean ± SEM. The quantitative data was compared separately between male and female groups using the unpaired Student's *t*-test. Due to the qualitative nature of the scoring, the Mann-Whitney test was applied to compare the pathology damage score between the groups. *P* value <0.05 was considered statistically significant.

## 3. Results

**Figure 1:**
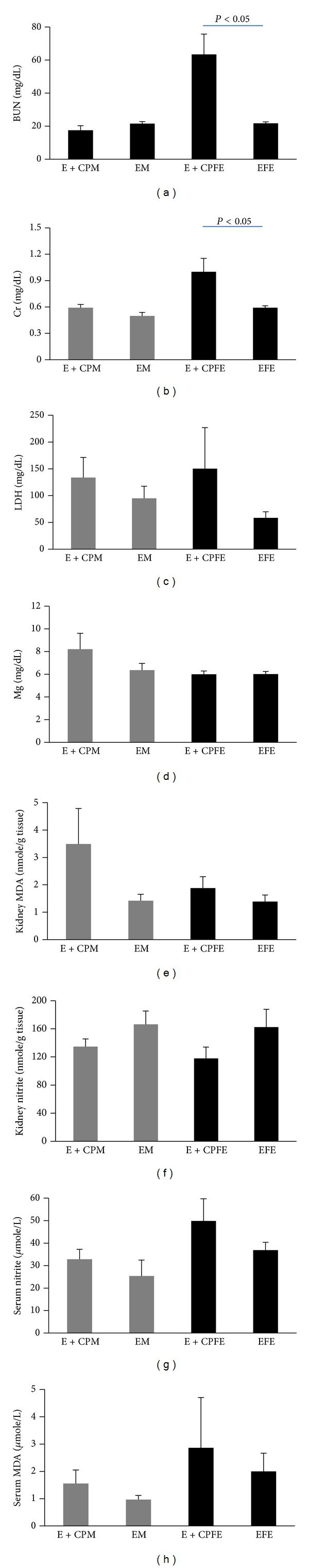
(a) Blood urea nitrogen (BUN), (b) creatinine, (c) lactate dehydrogenase (LDH), and (d) magnesium (Mg) levels in serum, (e) kidney tissue levels of malondialdehyde (MDA) and (f): nitrite, and serum levels of (g) nitrite and (h) MDA in four experimental groups of animals treated with CP and vitamin E. E + CPM, E + CPFE, EM, and EFE stand for the group names: males treated with vitamin E and CP, females treated with vitamin E and CP, males treated with vitamin E alone, and females treated with vitamin E alone, respectively.

**Figure 2:**
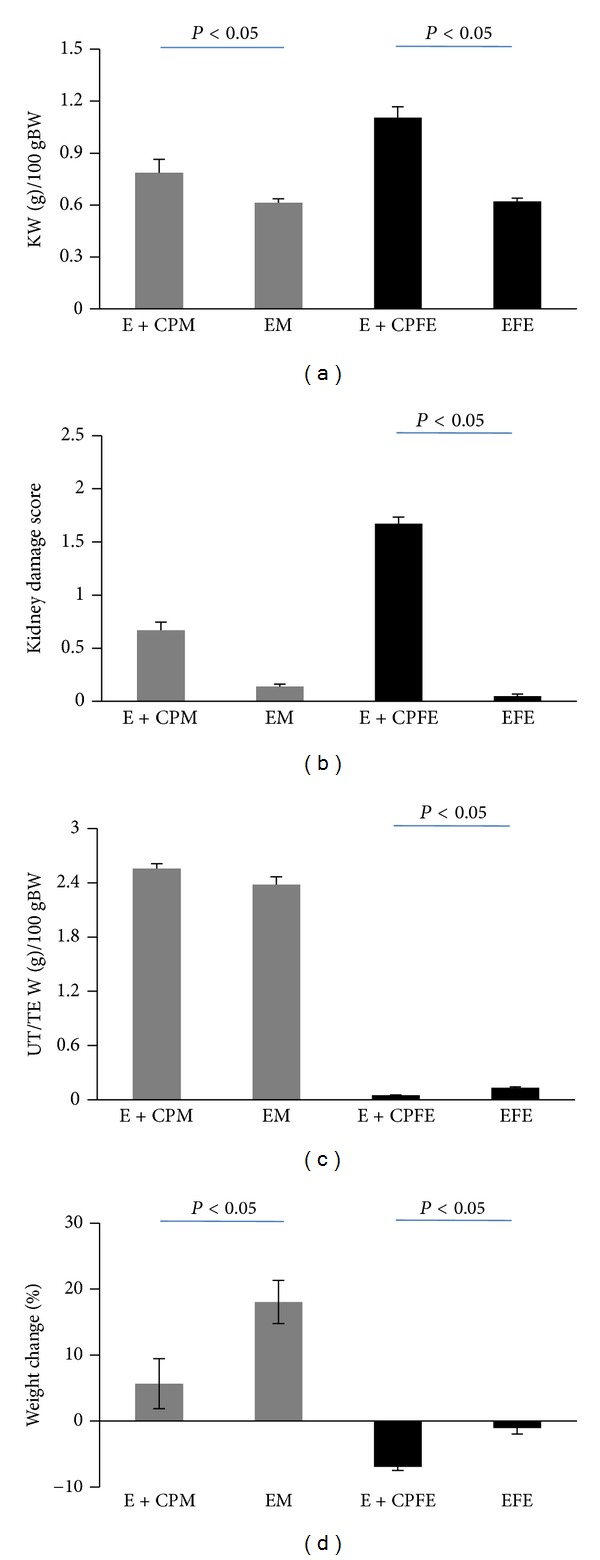
(a) Kidney weight (KW), (b) kidney damage score, (c) uterus/testis weight, and (d) percentage of weight change in four experimental groups of animals treated with CP and vitamin E. E + CPM, E + CPFE, EM, and EFE stand for the group names: males treated with vitamin E and CP, females treated with vitamin E and CP, males treated with vitamin E alone, and females treated with vitamin E alone, respectively.

**Figure 3:**
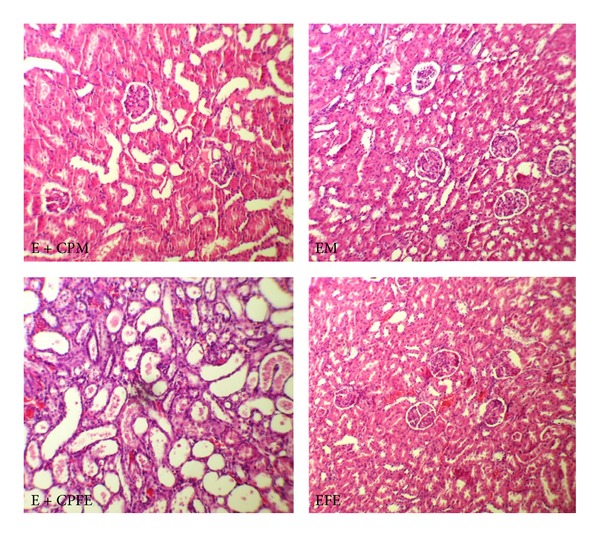
The images of kidney tissues (magnification ×100) in all groups of experiments. E + CPM, E + CPFE, EM, and EFE stand for the group names: males treated with vitamin E and CP, females treated with vitamin E and CP, males treated with vitamin E alone, and females treated with vitamin E alone, respectively. More kidney tissue damage was observed in groups E + CPFE.

The data are presented in Figures 1 and 2. The serum levels of BUN and Cr were significantly different in female (*P* < 0.05) but not in male. These findings indicated that vitamin E could not attenuate the increased serum levels of BUN and Cr in female. The pathology score data also reveals similar results, indicating the nephroprotective role of vitamin E in male but not in female (Figure 3). The normalized kidney weight and percentage of body weight change were significantly different in both genders (*P* < 0.05). However, vitamin E attenuated these data toward normal value in male more than female. No significant difference was observed in testis weight between the male groups, but the female groups were significantly different in the uterus weight (*P* < 0.05). No significant difference was observed in serum levels of Mg, LDH, nitrite, and MDA; and the kidney tissue level of nitrite and MDA was observed between the CP-treated and control groups in male or in female animals.

## 4. Discussion

The main objective of this research was to determine the role of gender in protective effect of vitamin E against CP-induced nephrotoxicity in the rat model. We showed the sex-related effects of vitamin E on CP-induced nephrotoxicity. CP reduced body weight in both male and female rats that may be related to disturbances in gastrointestinal or tubular absorption [26–28], as well as loss of skeletal muscle and apoptosis [27, 29]. The weight loss was also confirmed by our previous studies [21, 23]. Administration of vitamin E ameliorated weight loss induced by CP in male but not in female. In agreement with results obtained in the present study, we previously have reported that L-arginine and losartan as supplements ameliorate weight loss only in CP-treated male animals [21, 22]. Administration of vitamin E normalized the increased serum levels of BUN and Cr only in male animals, possibly due to the antioxidant effects of vitamin E against CP-induced nephrotoxicity mediated by oxidative stress. Vitamin E acts as free radical scavenger against stress oxidative [30]. CP induces oxidative renal damage, which results in production of free radicals and lipid peroxidation in tubular cells [5]. This was exhibited by nonsignificant elevated values of MDA in both sexes. Hypomagnesemia is one of the side effects of CP that occurs within two weeks after CP administration [31]. However, we did not observe Mg depletion in the current study, probably due to the duration of the study (one week). Histopathological investigations have demonstrated that CP administration reduces testis weight [32]. CP induces apoptosis in germ cells and Sertoli cells and also reduces the diameter of seminiferous tubules and serum level of testosterone [32]. Our findings showed that vitamin E prevented testicular weight reduction induced by CP, and another study also showed that different levels of vitamin E in diet affect the testis indexes [33]. An evidence showed that vitamin E can protect ley dig cells against reactive oxygen species [33]. Furthermore, our study demonstrated that vitamin E could not inhibit reduction of uterus weight induced by CP. CP caused apoptosis and necrosis in uterus tissue [34], and it seems that CP interacts with female hormonal system [35]. Recently, we provided an evidence for unfavorable effect of estrogen on nephrotoxicity induced by CP in ovariectomized female rats [23], and when estrogen was accompanied by antioxidants such as vitamins E and C and losartan, renal failure was intensified [23]. Also, our previous studies indicated that L-arginine and losartan as supplements induce different responses in nephrotoxicity model induced by CP in both male and female rats [21, 23]. Involved mechanisms are not exactly known, but it is clear that sex hormones play an important role in CP-induced nephrotoxicity in female. Female sex hormone also induces production of nitric oxide [36]. Nitric oxide, as a vasodilator agent, involved in kidney circulation [37]. Coadministration of CP and vitamin E nonsignificantly increased the serum level of nitrite in both genders. In contrast, vitamin E accompanied by CP nonsignificantly decreased the kidney level of NO in both genders. It seems that both renal and serum levels of NO were affected by various mechanisms. CP induces endothelial dysfunction and increases iNOS level [38], and NO involved in CP-induced toxicity [39]. Elevation of serum levels of NO by L-arginine enhanced CP-induced nephrotoxicity in female rats [21]. In this study, we observed elevated serum levels of nitrite in female more than male rats, probably due to the increasing iNOS level and presence of estrogen. It has been documented that estrogen itself induces oxidative stress [40] and promotes NO production.

## 5. Conclusion

It is concluded that vitamin E protects male gender against CP-induced nephrotoxicity. However, it fails to prevent CP-induced nephrotoxicity in female. Also interaction of supplements with gender difference is not known, and it requires further investigations.
